# A Dual Diagnosis of Okur–Chung Neurodevelopmental Syndrome and Becker Muscular Dystrophy: Inquiry Into the Lower Limits of Neurodevelopmental Functioning Attributable to Muscular Dystrophy

**DOI:** 10.1002/brb3.70276

**Published:** 2025-02-06

**Authors:** Victoria Liu, Eva Hanson, Joshua W Owens, Robert J. Hopkin, Amelle Shillington

**Affiliations:** ^1^ University of Cincinnati College of Medicine Cincinnati Ohio USA; ^2^ Division of Human Genetics Cincinnati Children's Hospital Medical Center Cincinnati Ohio USA; ^3^ Department of Pediatrics University of Cincinnati College of Medicine Cincinnati Ohio USA

**Keywords:** Becker Muscular Dystrophy, *DMD* gene, Duchenne Muscular Dystrophy, Okur–Chung Neurodevelopmental Syndrome

## Abstract

**Purpose:**

This case discusses the limits of neurodevelopmental functioning attributable to Duchenne's Muscular Dystrophy (DMD) dysfunction.

**Method:**

A 3‐year‐old male presented with global developmental delay, growth failure, and dysmorphic facial features. An SNP microarray revealed an interstitial duplication in exon 55 of DMD suggestive of Becker Muscular Dystrophy (BMD), but his degree of delays led to follow‐up exome sequencing revealing a pathogenic *CSNK2A1* variant diagnostic for Okur–Chung Neurodevelopmental Syndrome.

**Findings:**

Large cohorts predict a full‐scale IQ (FSIQ) of 88.3 ± 13.9 among all patients with BMD and 86.1 ± 15.0 among all patients with DMD, while variants impacting the brain dystrophin isoform Dp140 are associated with FSIQ of 77.7 ± 10.8 in BMD and 78.8 ± 18.6 in DMD.

**Conclusion:**

An FSIQ one standard deviation below these expected ranges should prompt screening for alternative causes of neurodevelopmental delays, and an FSIQ two standard deviations below these ranges should prompt broad‐spectrum genetic testing.

## Graphical abstract

This article shares an interesting case of a dual diagnosis and explores the expected cognitive abilities attributed to each diagnosis. It will be helpful to clinicians to think about when to consider a dual diagnosis in a patient with muscular dystrophy.

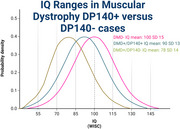

## 1 Introduction

Muscular dystrophies caused by pathogenic changes in the *DMD* gene, encoding the dystrophin protein, present as a spectrum of X‐linked recessive disorders (Darras et al. 2022; Nascimento Osorio et al. 2019). The more severe Duchenne Muscular Dystrophy (DMD) typically presents with delayed motor milestones in children younger than age 5, and by age 12 most children are wheelchair‐bound (Darras et al. 2022). Becker Muscular Dystrophy (BMD) has a milder presentation with later onset and progression of symptoms. In general, pathogenic variants in the *DMD* gene resulting in complete loss of function lead to a DMD phenotype, while variants with partial residual function lead to a BMD phenotype (Nascimento Osorio et al. 2019).

Muscular dystrophies are additionally associated with neurodevelopmental disorders, thought second to dystrophin's expression in the brain. The Dp427 *DMD* isoform is the full dystrophin protein and is disrupted in all pathogenic *DMD* variants. Variants downstream of exon 45 are expected to cause dysfunction of the Dp140 isoform, and variants downstream of exon 63 are expected to cause Dp71 dysfunction (Pascual‐Morena et al. 2023). These isoforms impact neurological function in various ways: Dp427 anchors GABA_a_ receptors to postsynaptic membranes of GABAergic neurons, Dp140 potentially contributes to early CNS dendrite development and neuronal differentiation (Vaillend and Chaussenot 2017), and Dp71 contributes to neuronal differentiation, cell adhesion, and excitatory synapse formation (Naidoo and Anthony 2020). Individuals with DMD on average have more severe neurodevelopmental symptoms compared to those with BMD (Hendriksen et al. 2020). Pathogenic DMD variants impacting brain‐specific isoforms cause higher rates of neurodevelopmental disability, with 12% of Dp427−/Dp140+/Dp71+, 29% of Dp427−/Dp140−/Dp71+, and 84% of Dp427−/Dp140−/Dp71− individuals experiencing intellectual developmental disorder (Pascual‐Morena et al. 2023).

Okur‐Chung Neurodevelopmental Syndrome (OCNDS) is an autosomal dominant disorder characterized by developmental delay, intellectual disability, dysmorphic facial features, generalized hypotonia, short stature, and autism spectrum disorder. OCNDS is caused by de novo pathogenic *CSNK2A1* variants, which encode the ubiquitously expressed alpha 1 catalytic subunit of protein kinase CK2 that has roles in cellular trafficking (Doray et al. 2002) and DNA repair (Loizou et al. 2004). Since first being characterized in 2016 (Okur et al. 2016), more than 35 individuals with OCNDS have been identified.

Here, we report a 3‐year‐old male with a known pathogenic variant in DMD who had developmental delays in excess of what could be attributed to muscular dystrophy, prompting additional genetic testing revealing a dual diagnosis of muscular dystrophy and OCNDS. We go on to identify the limits of expected intellectual functioning in muscular dystrophy to help providers recognize appropriate opportunities to pursue additional diagnostic workup.

## 2 Materials and Methods: Case Report

The patient is a 3‐year‐old male born to nonconsanguineous parents who presented with global developmental delay, postnatal growth failure, feeding difficulties, self‐injurious behaviors, and recurrent infections. Following a benign pregnancy, he was born to a G2P2002 26‐year‐old mother at 39w1d gestation via repeat c‐section. At birth, his weight was 3.6 kg (55%ile), height was 52.1 cm (79%ile), and head circumference was 34.9 cm (32%ile). He was discharged from the newborn nursery after 48 h.

By 18 months of age, he demonstrated progressive growth failure (height 72.6 cm, 2.6%ile; weight 8.7 kg, 0.2%ile; HC 46 cm, 8.3%ile) and global developmental delay that led to a multidisciplinary workup. A hearing evaluation suggested a mild bilateral conductive hearing loss attributed to recurrent otitis media that later required patulous eustachian tube placement, though his hearing was confirmed to be sufficient for speech development. A genetics evaluation was notable for hypertelorism with epicanthal folds, flat nasal bridge, mild cupid's bow lip, and mild generalized hypotonia. A chromosomal microarray revealed a maternally inherited 109 kb Xp21.1 interstitial duplication with breakpoints in exon 55 of DMD, arr[GRCh37] Xp21.1(31534187_31643598)x2. Confirmatory next‐generation sequencing was sent (Invitae Neuromuscular Panel), which confirmed a pathogenic duplication of exon 55 in DMD. Fragile X testing was negative.

At 23 months of age, The Developmental Profile‐Fourth Edition assessed his current developmental level as ranging from approximately 4 to 11 months (physical 8–9 months, adaptive behavior 10–11 months, social‐emotional 4–5 months, cognitive 8–9 months, and communication 6–7 months). He did not meet the criteria for autism. A brain MRI around this time demonstrated patchy abnormal hyperintense signals within the posterior white matter and subcortical white matter of the frontal lobes suggestive of a prior insult or ongoing demyelinating process. Multiple creative phosphokinase levels returned within normal limits. Due to his ongoing failure to thrive, he had a G‐tube placed at 24 months of age with subsequent improvement in his growth parameters and general well‐being.

## 3 Results

Given his dysmorphic features, excessive global delays than expected for muscular dystrophy, brain MRI abnormalities, and neuromuscular clinical findings more consistent with BMD than DMD, he returned to the genetics clinic for further evaluation. At 30 months of age, he received clinical exome sequencing that confirmed a de novo *CSNK2A1* c.593A > G (p.Lys198Arg) pathogenic variant consistent with a diagnosis of OCNDS. Following this diagnosis, he met with an endocrinologist who confirmed normal IGF‐BP3 (2250 ng/mL) and IGF‐I (99.2 ng/mL) levels, reassuring against a growth hormone deficiency. Given his history of recurrent upper respiratory infections and reports of previous immunoglobulin deficiencies in OCNDS, he had an immunology workup including a B cell and immunoglobulin workup returning as normal. An MRI of the pelvis and thighs using myopathy/myositis protocols at 33 months of age was normal, providing further evidence that the *DMD* variant was not the primary cause of gross motor delays.

He was most recently evaluated at 42 months of age and had a weight of 13.6 kg (13.6%ile), height of 91.1 cm (2.1%ile), and head circumference of 48.5 cm (∼8%ile). He receives the majority of his nutrition via a G‐tube, though he does tolerate small tastes. He continues to have global delays but has ongoing improvement in gross motor, fine motor, and communication skills. His neuromuscular exam at this time demonstrated mild hypotonia with normal ambulation, labs demonstrated normal creatine kinase levels.

## 4 Discussion

This case describes a male patient who presented with more severe developmental delays than expected for muscular dystrophy, and further diagnostic workup revealed a dual diagnosis of muscular dystrophy and OCNDS. This patient's small duplication affects exon 55 of the *DMD* gene, which would be expected to disrupt Dp140 while leaving Dp71 intact. Of note, prior publications have demonstrated that duplications in exon 55 of DMD can result in either a DMD or a BMD phenotype, with out‐of‐frame duplications likely contributing to a more severe phenotype (Juan‐Mateu et al. 2015). However, this patient's dysmorphic facial features, feeding intolerance, G‐tube dependence, hypotonia with a negative Gower's sign, normal CPK, and benign myopathy MRI suggested his presentation could not be due to muscular dystrophy alone. Developmental and language delay of varying severity have been universally reported in cases of OCNDS, and this patient's facial features of hypertelorism, epicanthal folds, and broad nasal bridge were frequently reported in other OCNDS cases (Okur et al. 2016). We thus conclude his presentation is heavily driven by the pathogenic *CSNK2A1* variant at this time, though we anticipate he may develop symptoms related to BMD in the future. Table 1 further compares the symptoms in this patient to symptoms from 36 previously reported OCNDS patients collected across 13 publications in the medical literature, in addition to describing expected symptoms in BMD and DMD. Additional OCNDS patient data are available in Table 1.

**TABLE 1:** Comparison of the reported patient to muscular dystrophy and OCNDS patients.

	Reported patient	OCNDS	BMD^a^	DMD^b^
**Sex**	Male	16/36 Male	Male	Male
**Age**	3 years 9 months	7.4 (mean)	Earlier onset	Later onset
**Craniofacial**				
Hypertelorism	Y	5/13	—	—
Epicanthal folds	Y	8/14	—	—
Arched eyebrows	N	9/18	—	—
Low‐set ears	N	7/14	—	—
Broad nasal bridge	Y	6/13	—	—
Anteverted nares	N	3/11	—	—
Microcephaly	N	10/34	—	—
Prominent forehead	N	4/14	—	—
Retrognathia	N	6/16	—	—
**Developmental**				
DD/ID	Y	36/36	+(Yaworski and McMillan 2020)	+(Mirski and Crawford 2014)
Speech present	Y	26/30	+	+
ADHD	NA	2/4	+(Yaworski and McMillan 2020)	+
**Neurologic**				
Seizure	N	10/24	—	—
Hypotonia	Y	20/26	—	—
Swallowing difficulties	Y	6/17	—	—
**Musculoskeletal**				
Joint hyperextensibility	NA	4/9	?	+(Ryder et al. 2017)
Scoliosis	N	2/8	?	+(Abbott et al. 2020)
**Cardiovascular disease**	N	4/14	++(Yaworski and McMillan 2020)	+++
**Gastrointestinal/Growth**				
Short stature	Y	22/23	—	—
Failure to thrive	Y	4/10	—	—
Feeding intolerance	Y	10/13	—	—
Require NG/GT	Y	3/7	—	—
Constipation	Y	3/6	—	—
Reflux	N	3/8	—	—
**Immunologic**				
Hypogammaglobulinemia	N	4/6	—	—
IgA deficiency	N	2/6	—	—
IgG deficiency	N	3/6	—	—

*Note*: Y = feature present; N = feature absent; NA = non‐applicable due to age;? = data unavailable; — = Feature in < 10% of patients; + = feature in 10%–50% of patients; ++ = feature in > 50% of patients; +++ = Universal feature.

^a^ Data from Dystrophinopathies GeneReviews unless cited.

^b^ Features not intended to describe end‐stage disease.

Both BMD and DMD are recognized to negatively impact full‐scale IQ (FSIQ) as measured by the Weschler Intelligence Scale, with large cohorts showing FSIQ of 88.3 ± 13.9 in patients with BMD and 86.1 ± 15.0 in patients with DMD (Szabo et al. 2022). Multiple studies have demonstrated that genetic changes that alter the dystrophin Dp140 and Dp71 isoforms found in the brain have more significant impacts on learning and development than variants that spare these isoforms. Previous studies comparing Dp140+ and Dp140− in BMD and DMD have established expected FSIQ ranges in these individuals (Table 2) (Okur et al. 2016; Szabo et al. 2022; Chamova et al. 2013). Patients who were Dp71− had worse neurodevelopmental outcomes (Iskandar et al. 2022), though there was insufficient data to establish expected IQ ranges.

**TABLE 2:** Expected IQ ranges in BMD/DMD, with lower limits to consider investigating other conditions.

	Isoform	Reported patients	Mean full‐scale IQ^a^	Standard deviation	Lower limit IQ to screen for additional diagnosis (< 1 SD)	Lower limit IQ to perform additional genetic testing (< 2 SD)
**BMD**	All	85	88.3	13.9	74.4	60.5
	DP140+	9	88.5	8.94	79.6	70.6
	Dp140−	4	77.7	10.8	66.9	56.2
**DMD**	All	513	86.1	15.0	71.1	56.1
	DP140+	118	91.3	17.1	74.2	57.1
	Dp140−	224	78.8	18.6	60	41.6

^a^ As measured by Weschler Intelligence Scale.

Using these established ranges, we propose that any patient with a diagnosis of BMD/DMD with an FSIQ one standard deviation below the expected mean should be screened for features suggestive of an additional diagnosis, though their Dp140 status should be considered when determining expected intellectual functioning. If at least one congenital anomaly or unexpected symptom not explained by muscular dystrophy is detected, the patient should undergo further diagnostic workup targeting their delays and other symptoms. If the patient's FSIQ is greater than two standard deviations below the established mean, they should receive exome or genome sequencing for investigation of DD/ID (Manickam et al. 2021) regardless of whether additional anomalies are present. As a more generalizable statement, patients with Dp140+ muscular dystrophy with borderline intellectual functioning should receive screening for a potential second diagnosis contributing to their neurodevelopmental symptoms, while those with an established mild intellectual disability should receive additional genetic workup. For Dp140− patients, those with mild intellectual disability should be screened for a second diagnosis and those with a moderate intellectual disability should receive additional genetic testing.

## 5 Conclusion

In conclusion, we report the first recognized case of a dual diagnosis of OCNDS and muscular dystrophy. Identifying excessive developmental delays and features not attributable to muscular dystrophy should prompt a workup for additional diagnoses impacting neurodevelopment.

## Author Contributions

**Victoria Liu**: writing–original draft, writing–review and editing, data curation**. Eva Hanson**: writing–original draft, writing–review and editing, data curation. **Joshua W Owens**: conceptualization, writing–original draft, writing–review and editing, supervision. **Robert J. Hopkin**: writing–review and editing, project administration, supervision**. Amelle Shillington**: writing–review and editing, project administration, supervision.

## Disclosure

We confirm that we have read the Journal's position on issues involved in ethical publication and affirm that this report is consistent with those guidelines.

## Consent

The patient's parents have provided written consent for publication following their review of this manuscript.

## Conflicts of Interest

The authors declare no conflicts of interest.

### Peer Review

The peer review history for this article is available at https://publons.com/publon/10.1002/brb3.70276.

## Data Availability

All data used in the creation of this manuscript is available upon request.
